# Screening for cardiac sarcoidosis: diagnostic approach and long-term follow-up in a tertiary centre

**DOI:** 10.1007/s12471-025-01994-9

**Published:** 2025-10-06

**Authors:** Beverly I. de Leeuw, Harold Mathijssen, Marco C. Post

**Affiliations:** Department of Cardiology, St. Antonius Hospital, Nieuwegein, The Netherlands

Dear Editor,

Van der Velde and colleagues conducted a retrospective study investigating a diagnostic approach with its long-term outcomes in sarcoidosis patients with suspected cardiac involvement at their tertiary centre [1]. The approach aligned with the Heart Rhythm Society’s recommended algorithm, utilizing history, electrocardiography (ECG), and transthoracic echocardiography (TTE) as initial screening tools prior to cardiac MRI or PET/CT [2].

In the study, absence of cardiac symptoms and normal ECG and TTE findings did not exclude cardiac sarcoidosis (CS). In fact 38% of asymptomatic patients and 13% of patients with normal ECG and TTE were ultimately diagnosed with CS. However, not all patients underwent cardiac MRI, despite its reported excellent diagnostic and prognostic value [3]. This raises several questions: Under what conditions should CS screening be initiated, if symptoms alone are not discriminative? How much diagnostic weight should be given to ECG and TTE? Interestingly, patients with normal ECG and TTE showed favorable prognoses regardless of CS diagnosis. Given the limited treatment implications, could this information be sufficient to avoid further testing? If not, more robust screening tools—such as speckle-tracking echocardiography—should be considered.

## References

[1] Van der Velde N, Poleij A, Lenzen MJ, et al. Screening for cardiac sarcoidosis: diagnostic approach and long-term follow-up in a tertiary centre. Neth Heart J. 2025;33:55–64. 39786690 10.1007/s12471-024-01925-0 PMC11757833

[2] Birnie DH, Sauer WH, Bogun F, et al. HRS expert consensus statement on the diagnosis and management of arrhythmias associated with cardiac sarcoidosis. Heart Rhythm. 2014;11:1305–23. 24819193 10.1016/j.hrthm.2014.03.043

[3] Athwal PSS, Chhikara S, Ismail MF, et al. Cardiovascular Magnetic Resonance Imaging Phenotype and Long-term Outcomes in Patients With Suspected Cardiac Sarcoidosis. JAMA Cardiol. 2022;7:1057–66. 36103165 10.1001/jamacardio.2022.2981 PMC9475438

